# The effect of *fs800* on female egg production in *Schistosoma mansoni*

**DOI:** 10.1016/j.molbiopara.2021.111412

**Published:** 2021-09-04

**Authors:** Sevan N. Alwan, Philip T. LoVerde

**Affiliations:** a Departments of Biochemistry and Structural Biology, UT Health at San Antonio, San Antonio, TX 78229, USA; b Pathology and Laboratory Medicine, UT Health at San Antonio, San Antonio, TX 78229, USA

**Keywords:** *Schistosoma mansoni*, *fs*800, Eggs, Schistosomiasis, RNAi

## Abstract

During schistosomiasis, the paired *Schistosoma mansoni* female produces about 300 eggs each day. These eggs are responsible for the clinical picture and the transmission of the disease. During female development and egg production, *fs800* is expressed only in female vitelline cells. Blast search of *fs800* did not show similarities with any published sequences by NCBI. We hypothesize that the product of this gene plays a role in *S. mansoni* egg production. By using RNA interference to knockdown f*s800* and quantitative PCR to measure the gene expression in the female schistosomes, we were able to demonstrate that *fs*800 product is crucial for viable egg production, it has no effect on worm health or male-female pairing. Our data suggest *fs800* inhibition as a potential target to prevent transmission and pathology of schistosomiasis.

## 1. Introduction

Human schistosomiasis, a neglected tropical disease, is caused by three major human species *Schistosoma mansoni*, *S. haematobium* and *S. japonicum*. Schistosomiasis affects some 229 million people in 78 countries. It is ranks with malaria as one of the most devastating parasitic diseases in terms of impact [1–4].

Infection with *Schistosoma mansoni*, an intravascular parasite, gives rise to hepatic and intestinal schistosomiasis. Unlike most trematodes, schistosomes exhibit separate sexes. An interesting interplay between the male and female has evolved as a result [4].

Female schistosomes do not mature physically or reproductively unless they pair with the male. Female worms from single-sex infections are underdeveloped in that they are stunted and have an immature reproductive system compared to female worms from a paired infection. Upon pairing with a male worm, the female’s sexual organs become mature, and egg production begins. However, if the female is widowed, she will regress to the immature state until she is remated with a male worm [5,6]. Thus, the male sends an unknown signal to initiate and maintain female reproductive development.

As a result, each worm pair produces around 300 eggs per day [7]. Approximately half of these eggs reach the gut lumen and are released with feces into the environment. The other half swept up in the circulation are trapped in the tissues leading to a granulomatous inflammatory response. The symptoms of schistosomiasis result from the host’s inflammatory reactions against the trapped eggs in the tissues [8]. Currently there is no effective vaccine against schistosomes, the only treatment available for human schistosomiasis is praziquantel. As a monotherapy in Mass Drug Administration (MDA), resistance against praziquantel has become a serious concern [9,10].

Differentiation, maturation, and maintenance of the female reproductive system requires continuous male-female pairing. This interplay between male and female worms results in a series of changes that include differentiation of germ cells in females into vitellocytes [11–14]. Several genes have been characterized during this process [15–18]. It turns out that some of these genes such as p14 and p48 are expressed in a stage (mated female), tissue (vitelline cell) and temporal (upon pairing with the male) specific manner [19]. Female specific 800 gene (*fs800*) is one of these genes that is expressed only in female vitelline cells in response to pairing with the male resulting in reproductive maturation and egg production [20,21]. The purpose of this study is to investigate and understand the role of *fs800* gene products in *S. mansoni* egg production. Targeting the egg stage may offer another drug or vaccine target to reduce morbidity and prevent transmission.

## 2. Material and methods

### 2.1. Parasite maintenance

*S. mansoni* was maintained by passage through a snail intermediate host, *Biomphalaria glabrata,* and golden Syrian Hamsters as a definitive host [22]. Hamsters were infected with 250–350 cercariae for parasite maintenance according to IACUC protocol (Protocol #08039).

### 2.2. Parasite recovery

Animals infected with *S. mansoni* were sacrificed after 20, 29, 36, and 45 days post-infection (dpi). In accordance with IACUC protocol (UTHSCSA IACUC Protocol #08039), animals were euthanized by intraperitoneal injection using Fatal-Plus (Butler Animal Health, Ohio), a sodium pentobarbital solution, and 10 % heparin. Adult worms were collected by perfusion as previously described [23] using 0.9 % saline containing EDTA.

### 2.3. Worm in vitro culture

Worms were cultured in 2 ml 1X Dulbecco’s Modified Eagle Medium (DMEM, Gibco) with 10 % Heat Inactivated Fetal Bovine Serum (FBS, Atlantic Biologicals) and 1X antibiotic/antimycotic (Ab/Am, GIBCO). Worms were manually sorted under a dissecting stereomicroscope and aliquoted to 10 paired worms per well in a 6-well plate. Worms were treated with *fs800* dsRNA right after sorting. Worms collected after 20 days of infection did not show gender differences and therefore were not sorted by sex. Worms were kept in an incubator at 37 °C and 5% CO_2_ for 72 h. Worm viability in culture was assessed by daily observation for three days [24].

### 2.4. RNAi interference

Primers amplifying a 303 bp section of the gene coding region were designed using the Primer-Blast tool by NCBI, forward primer 5′-CTCGTGGAAATAGTGCAAAAGG-3 ‘and reverse primer 5^′^-GTCGACCA-TATCTATCTGTTCC-3’. Polymerase chain reaction (PCR) was performed to produce an amplicon, followed by confirmation of amplification by running the PCR product on a 1 % agarose gel. Then T7 promoters were added to the forward and reverse primer to flank the PCR product. Confirmation of amplification was also performed *via* 1 % agarose gel. The PCR product with T7 promoters were used as a template for transcription of the dsRNA. The dsRNA was placed in a 37 °C water bath overnight, then treated with DNAase to remove contaminants. Ammonium acetate (3 M) was added, followed by 100 % ethanol to precipitate the RNA. The RNA was left at this step overnight. Then the sample was centrifuged at 14,000 rpms, forming an RNA pellet. The pellet was washed twice with 70 % ethanol. On the second wash, the supernatant was removed, and the ethanol allowed to evaporate. Then the pellet was resuspended in nuclease-free water. The concentration of RNA was then measured using the Thermo Scientific NanoDroprop 1000 spectrophotometer.

Ten worms pairs collected at 29, 36 and 45 days post-infection were treated with 50 μg/mL dsRNA in triplicate of either *S. mansoni fs800* (Smp_307900) or irrelevant control *Luciferase* (M15077) or the *Drosophila nautilis* gene (U23431) right after worm sorting. Pilot study showed that 30 μg/mL dsRNA to knockdown *Schistosoma* genes, only resulted in 74 % reduction in viable egg production. Increasing the amount of dsRNA to 50 μg/mL resulted in about 90 % reduction in viable egg production. Worms were observed 3 days for worm health, viability, and pairing. The worms were sorted into males and females. All males were quick-frozen. Half of the females were quick-frozen and the other half were fixed in 10 % neutral buffered formalin overnight embedded in paraffin, sectioned and stained with Hematoxylin and Eosin by STRL UTHSCSA Histology/ Immunohistochemistry Laboratory, UT Health at San Antonio.

### 2.5. Eggs

*S. mansoni in vitro* cultured eggs were collected from control and *fs800* knockdown from 29, 36 and 45 day-old worms. Viable, non-viable and abnormalities of non-viable eggs were determined according to appearance and the ability of trypan blue to differentiate between the viable and non-viable eggs under light microscopy at 40X [27]. Eggs were counted by hemocytometry (Hausser Scientific, USA).

### 2.6. RNA extraction

Total RNA was obtained from frozen samples of 20, 29, 36 and 45 days of *S. mansoni* worms. All frozen samples were thawed on ice in RNAzol^®^RT (Molecular Research Center Inc.) each sample then was placed in 2 ml tubes of Lysin Matrix Tubes containing 1.4 mm ceramic spheres and then homogenized using Beadbeater homogenizer (Biospec, USA) for 45 s 2 ×. RNA was extracted and purified according to (Molecular Research Center Inc.) manufacturer instructions for total RNA isolation.

### 2.7. cDNA synthesis

cDNA was generated from total RNA using BioRad iSCRIPT cDNA Synthesis Kit according to the manufacturer’s instructions.

### 2.8. Quantitative real time PCR

Quantitative Real Time PCR (qRT-PCR) was used to determine the relative quantities of *fs800* transcribed in *fs800* RNAi treated worm pairs from 20, 29, 36 and 45 dpi. Three μL of cDNA was used for relative quantification with gene specific primers and iTaq^™^ Universal SYBR^®^ Green Supermix (BioRad) containing hot-start iTaq DNA polymerase, dNTPs, MgCl2, SYBR^®^ Green I dye, and ROX reference dye. Primers were designed using Primer-Blast tool by NCBI, forward primer 5^′^-CTCGTGGAAATAGTGCAAAAGG-3 ‘and reverse primer 5^′^-GTCGACCA-TATCTATCTGTTCC-3’. GAPDH (XM_018794048.1) was used as an endogenous control for knockdown groups and controls. The qRT-PCR reaction was performed in 10 μL reaction and contained 5 μl iTaq Universal SYBR^®^ Green Supermix (BioRad), 3 μl cDNA (50 μg/μl), 1 μl each of forward 5′- GTG AAA GAG ATC CAG CAA ACA T −3′ and reverse 5′- ATA TGA GCC TGA GCT TTA TCA ATG-3′ primers 10 μM. The qRT-PCR profile was 55 °C for 2 min, 98 °C for 10 min, 40 cycles of 98 °C for 15 s, 60 °C for 1 min, and a final step of 60 °C for 5 min (Applied Biosystems 7500 FastReal-Time PCR System).

Comparative *fs800* expression between S. *mansoni* knockdown group and control was calculated based on qRT-PCR amplification results.

### 2.9. In silico analysis

The following public domain tools were used for sequence and transcriptome analyses: NCBI-BLAST (http://blast.ncbi.nlm.nih.gov/Blast.cgi), http://schisto.xyz/ for genome version 5 and 7. fs800 is now designated Smp_307900 which is derived from two previous gene designations, Smp_000290 and Smp_077900 [28,29]. Transcriptome information was obtained from genome version 5 (http://schisto.xyz/, “Gene expression atlas in adult S. mansoni”), and SchistoCyte Atlas (http://www.collinslab.org/schistocyte/search?gene=Smp_307900 [29].

### 2.10. Statistical analyses

Statistical analyses were performed using GraphPad Prism software version 9.0.0(86). Multiple unpaired t tests were performed to compare absolute values of three individual experiments with identical conditions. Fraction of Total was used for parts-of-whole data.

## 3. Results

**Fig. 1.**
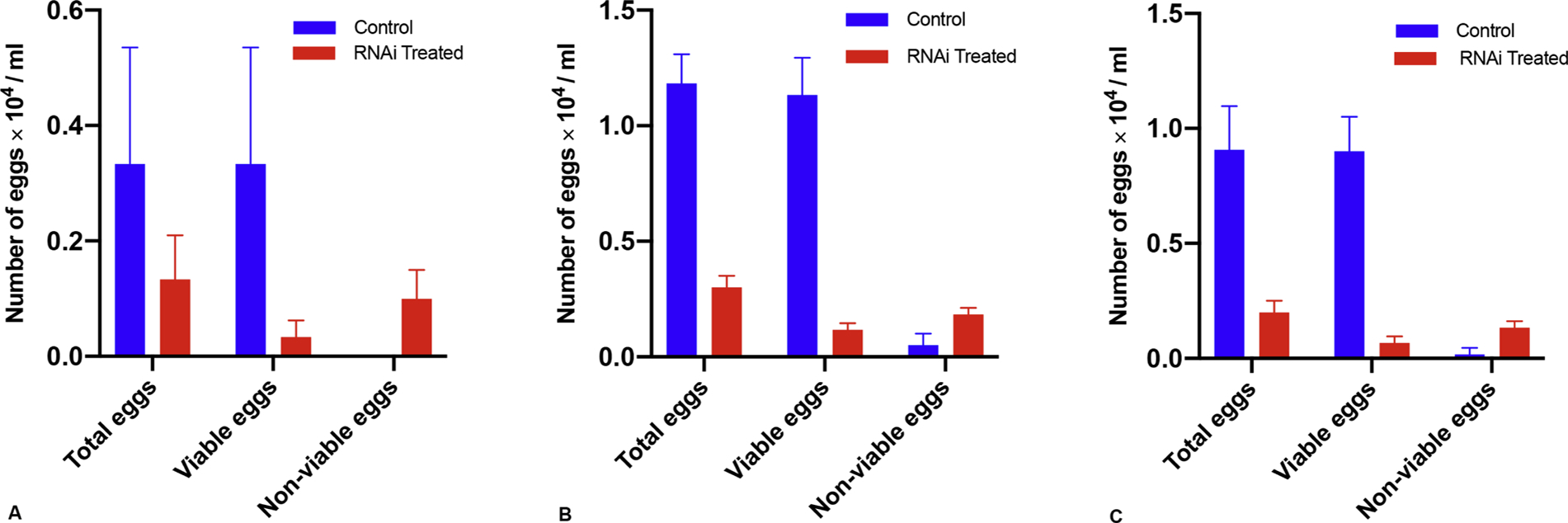
Number of total eggs, viable eggs, and non-viable eggs in culture media collected after 72 h. from *Smfs800* knockdown worms compared to the control. **A**. Eggs collected from 29 dpi, no statistical difference in the number of total eggs, viable eggs, and non-viable eggs compared to the control; *P values* were *0.184074*, *0.063603*, and *0.025721* respectively. **B**. Eggs collected from 36 dpi, statistical difference in the number of total eggs, viable eggs and non-viable eggs compared to the control *P values* were *0.000350*, *0.000419*, and *0.016130*, respectively. **C.** Eggs collected from 45 dpi, statistical difference in the number of total eggs, viable eggs and non-viable eggs compared to the control *P values* were 0.003387, 0.000700, and 0.007763, respectively. Data were analyzed by Multiple unpaired t test. Bar plot data by Mean with SD. Irrelevant *control was luciferase* (M15077).

**Fig. 2.**
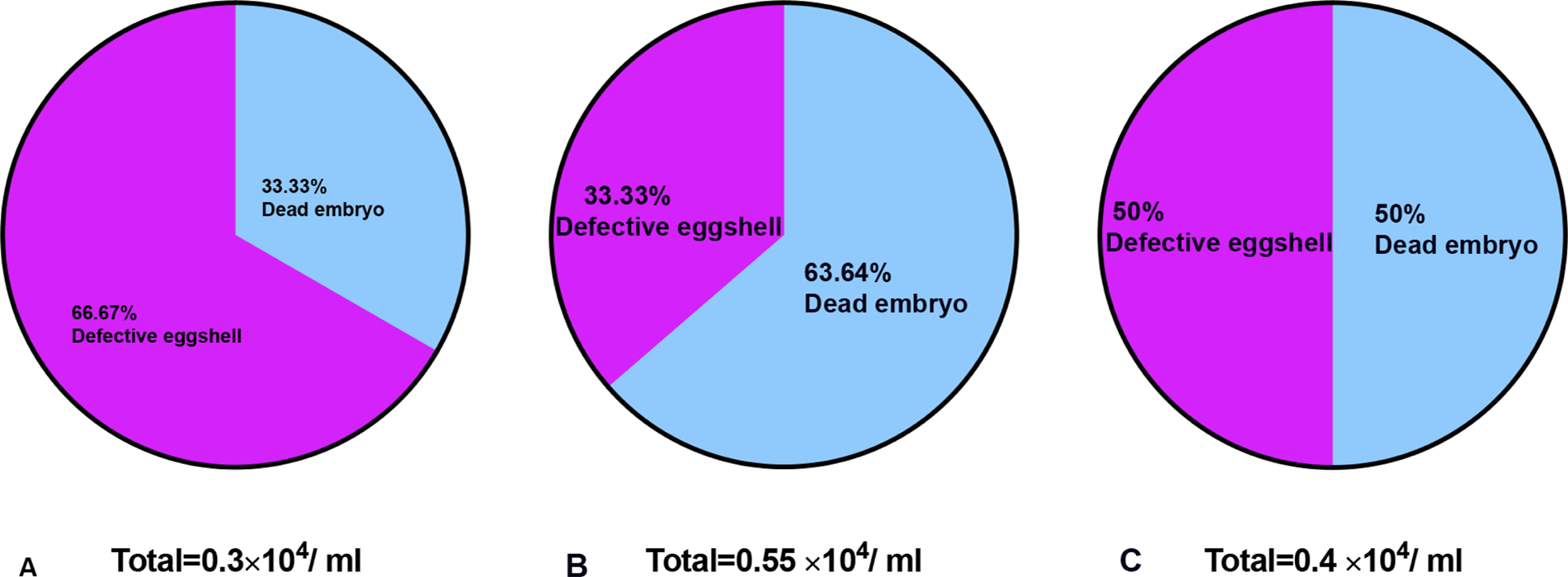
The percentages of defective eggshells and dead embryos among non-viable eggs collected after 72 h from *Smfs800* knockdown worms in culture media. **A**. Eggs collected from 29 dpi. **B**. Eggs collected from 36 dpi. **C.** Eggs collected from 45 dpi. The eggs were counted by hemocytometry. Egg viability was determined according to appearance using trypan blue under light microscopy at 40 ×. Data were analyzed by Fraction of Total.

Female schistosomes have migrated to the portal system of the liver and are morphologically undifferentiated at day 20 post-infection. At around 29 dpi, male and female worms begin to mate and move against the flow of blood to the mesenteric veins [15]. At 32–36 dpi, worm pairs begin to produce eggs. Schistosome development is asynchronous but at 45 dpi most of the males and females have mated and produce 300 eggs per day [7,15]. The results of egg production from female worms following *fs800* knocked down is shown in Fig. 1. At day 29 pi, the time mating begins there is no significant difference in the number of eggs produced by RNAi treated worms *versus* control worms. However, at day 36 and 45 there is a significant difference. The reduction in the number of viable egg production collected after 72 h after RNAi treatment on days 36 and 45 was 89.7 % and 92.6 %, respectively. If we just count the number of non-viable eggs from the total number of eggs produced and quantify those that have a defective eggshell or dead embryo (supplemental Table1), from day 29 to 45 post exposure the percentages of non-viable eggs with defective eggshells is 66.6 %, 33.33 % and 50 %, respectively as shown in Fig. 2.

**Fig. 3.**
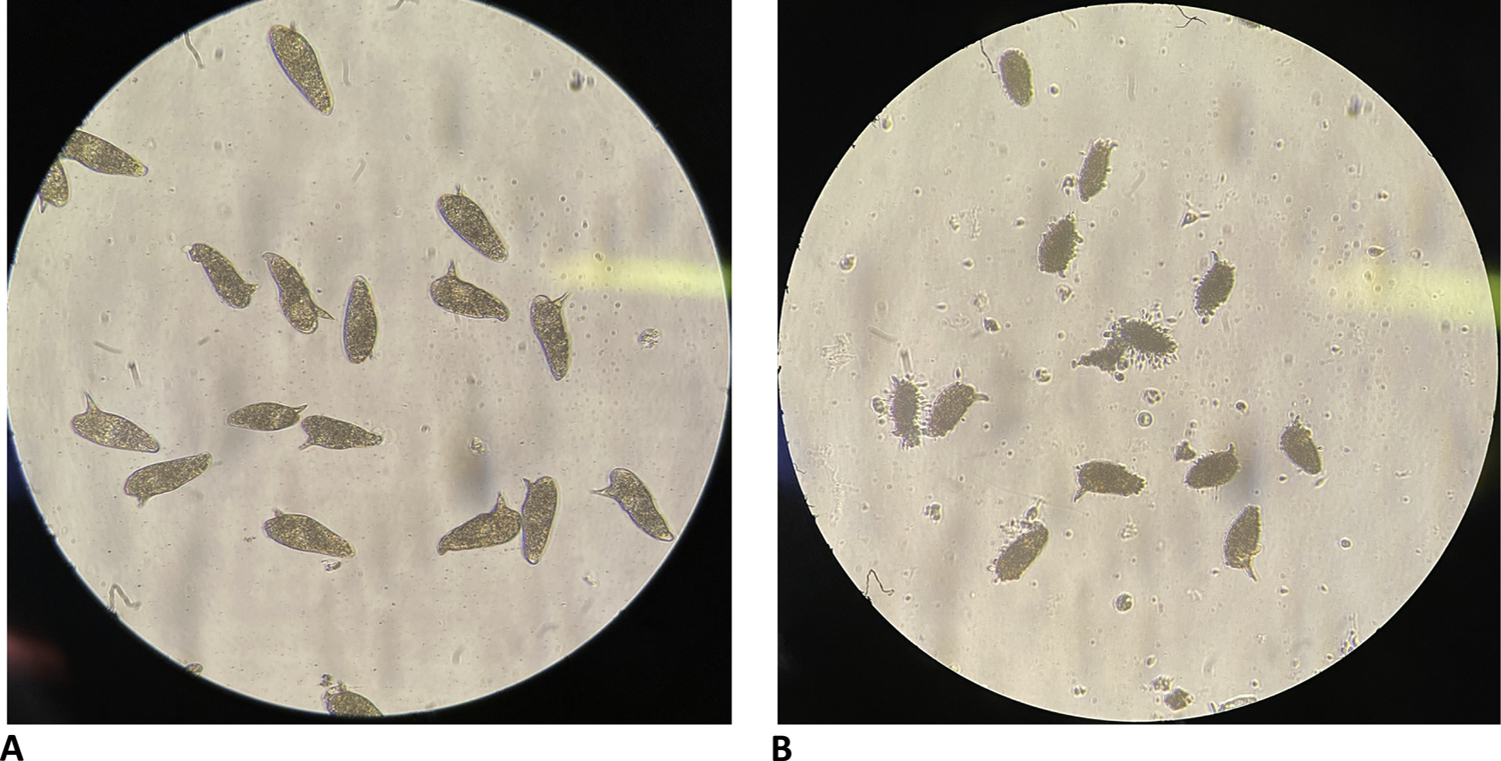
Eggs collected from *in vitro* cultured 45 dpi worm pairs under light microscopy at 40 ×. **A.** Eggs collected from control. **B** Egg collected from *Smfs800* knockdown. These photographs are representative of cultures from knockdown worms at 29, 36, and 45 dpi.

Fig. 3 shows representative eggs from 45 dpi control female worms compared to *fs800* knockdown worms. In the knockdown worms, a spike or spicule protruding phenotype can be observed along with eggshell fragments. This phenotype of eggs from worms that have experienced *fs800* knockdown is observed at 29, 36 and 45 dpi (Fig. 3). Videos of defective eggs from 45 dpi *fs800* knockdown worms and control female worms are shown in supplementary Fig. 1.

**Fig. 4.**
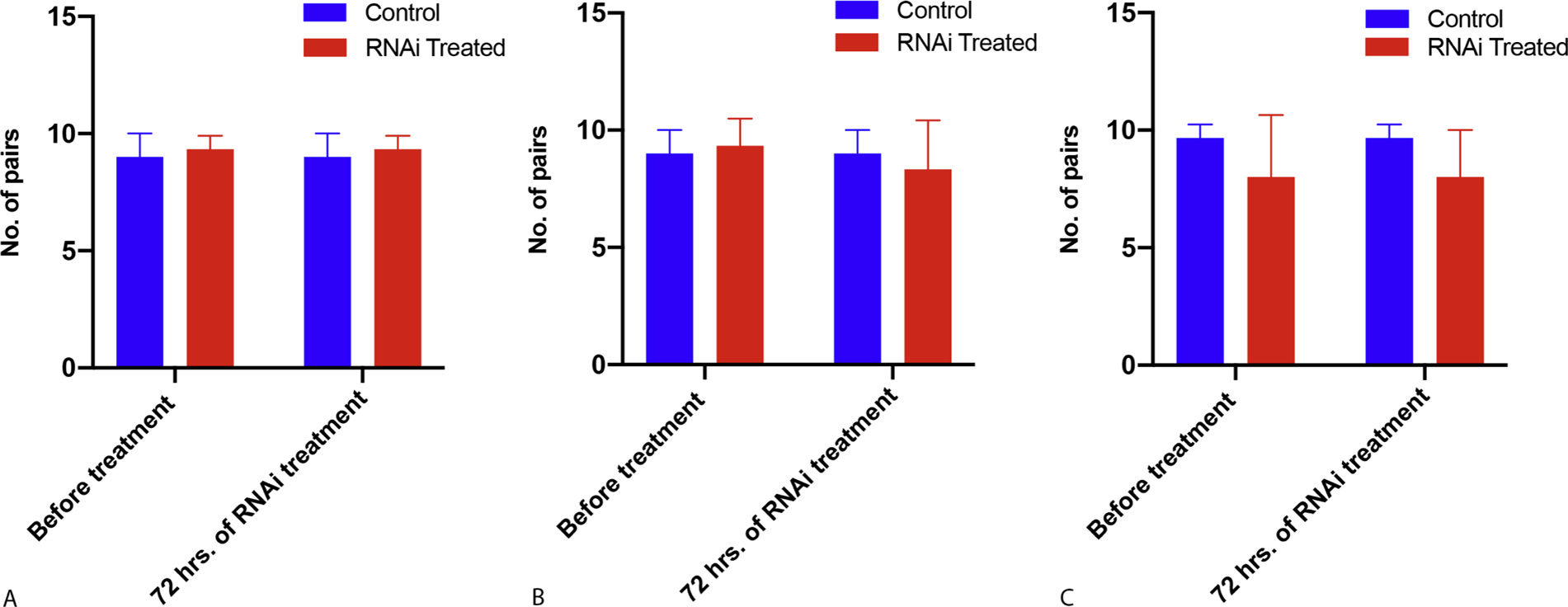
Number of male-female pairs in culture media after 72 h from *Smfs800* knockdown worms compared to the control. **A**. Number of pairs from 29 dpi, no statistical difference in pair numbers before and after knockdown compared to the control *P values* were *0.643330* and *0.643330,* respectively. **B**. Number of pairs from 36 dpi, no statistical difference in pair numbers before and after knockdown compared to the control *P values* were *0.724659* and *0.643330,* respectively. **C.** Number of pairs from 45 dpi, no statistical difference in pair numbers before and after knockdown compared to the control *P values* were *0.346490* and *0.237796*, respectively. Data were analyzed by Multiple unpaired t test. Bars plot data by Mean with SD.

We also addressed the question of whether *fs800* played a role in male-female mating. It turns out that knockdown of *fs800* had no significant effect on worm pair mating (Fig. 4).

**Fig. 5.**
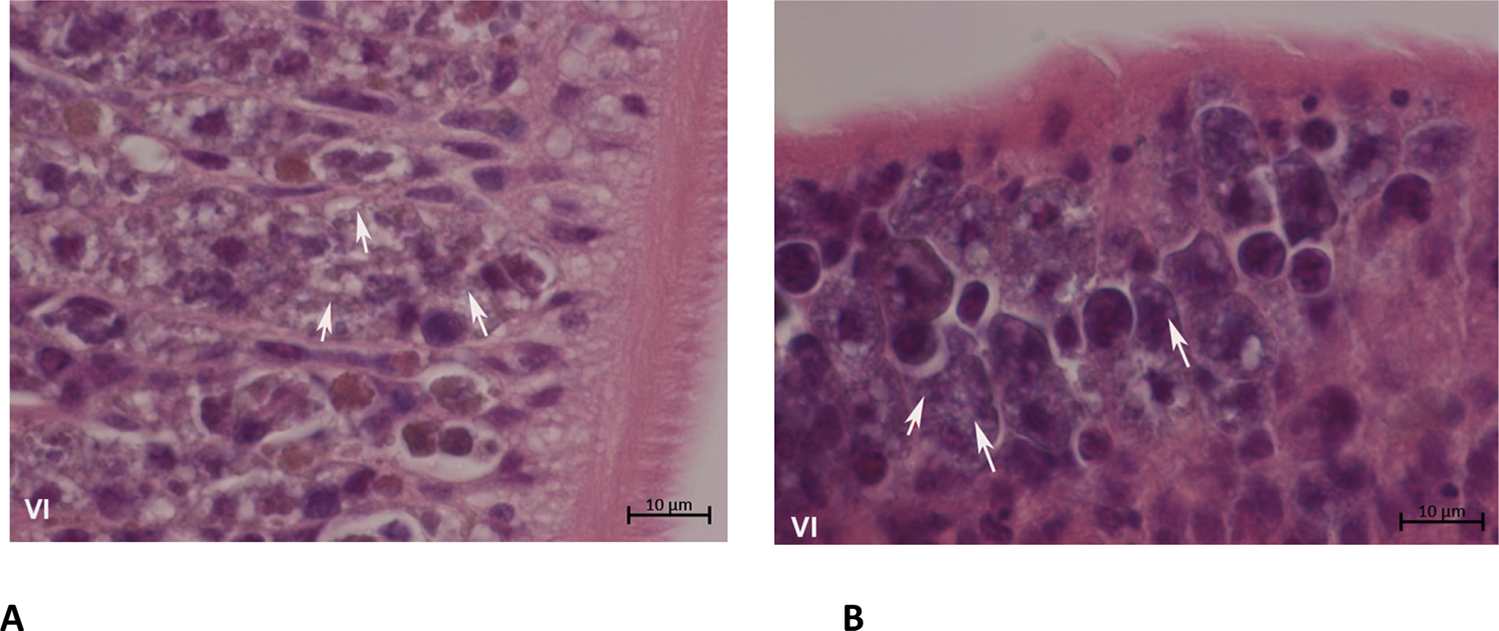
Z-stack images for sections represent the female vitellaria stained with H&E under Zeiss Axio imager Z1 & microscopy at 100 ×. **A.** Vitellaria from the *Smfs800* knockdown female. **B.** Vitellaria from a control female. *Abbreviation*: VI; Vitellaria, arrow heads, vitelline cell droplets.

Comparison of the ovaries from *fs800* knockdown worms and control female worms demonstrated no significant morphological differences. This was consistent with the literature [14,28,29]. However, the vitellaria from *fs800* knockdown worms showed a lack of development and maturation of vitellocytes. In addition, there was an apparent reduction in the number of vitellocytes compared to control female worms (Fig. 5A and B). It also appeared that many of the vitellocytes from fs800 knockdown worms had lost their cytoplasmic droplets compared to control treated worms (Fig. 5C and D).

## 4. Discussion

*Schistosoma* eggs are the primary agent of pathogenesis and responsible for transmission [8]. There is no effective vaccine against human schistosomiasis. Only one method of treatment, the drug praziquantel is used for control. However, it does not prevent reinfection and resistance is an emerging concern [9,10]. Without formation of the eggs there is no pathogenesis and there is no transmission. Therefore, understanding the molecular basis for female reproductive development and egg production may offer a target for drug or vaccine development to reduce morbidity and prevent transmission.

Schistosomes have evolved an interplay between male and female worms such that a male signal independent of sperm transfer regulates female gene expression [5]. Therefore, numerous studies have been focused on analyzing the changes in gene expression and localization of these genes during female maturation and egg production. *P14, p48, FS800*, and *S. mansoni tyrosinase 1* and *2* are among the genes expressed in female vitelline cells during egg production [16,19,20,31]. Our laboratory previously characterized *p14* and *p48* when we were able to show that the amino acid composition of the *p14* and *p48* product is fairly similar to the amino acid composition obtained from the hydrolysis of purified eggshells suggesting that *p14* and *p48* products are eggshell precursors [19,31]. In addition, immunoblots with anti-p14 antibodies demonstrated that p14 protein was present in the eggshell [32]. Likewise, the p48 protein was demonstrated to be associated with the egg by immunological means [33]. By using northern blot and *in situ* hybridization Reis et al. and others showed that *fs800* is expressed during maturity when *S. mansoni* females produce eggs and *fs800* mRNA was detected only in vitelline cells [20]. However, there is no data to date to indicate that the fs800 protein is present in the egg. However, we do know that the *fs800* RNA is expressed in the vitelline cells which are present in the egg. A study by Fitzpatrick et al. (2007) characterized *S. mansoni* tyrosinases 1 and 2 and showed that the diphenol oxidase enzyme activities are needed for eggshell formation. The same study suggested *S. mansoni* tyrosinase 1 as a therapeutic target.

These studies were followed by several studies that utilized different technologies such as combining laser microdissection and microarray to detect and localize the changes in gene expression in the tissues of adult females of *S. japonicum* [34], RT-PCR reactions and *in situ* hybridizations in *S. mansoni* [17], tissue-specific RNA-seq analyses of female ovaries and male testes of *S. mansoni* [35], RNA-seq analysis and *in situ* hybridizations to characterize the vitellarium of adult females of *S. mansoni* [36]. The results confirm the stage, tissue and temporal specific manner of expression of female-specific genes that were previously characterized in response to the male stimulus.

In the present study the level of *fs800* expression (supplementary Fig. 1) is consistent with previous studies [20,29,34–36] and with the recent single-cell dataset [29]. Our data show a significant reduction in the number of eggs produced in knockdown paired females 36 dpi and 45 dpi compared to the control group (Fig. 1) while a non-significant reduction was obtained from RNAi treated group in 29 dpi compared to the control group. Although, *S. mansoni* male and female parasites pair up after approximately 28 dpi [36], asynchronous development of *Schistosoma* could be a reason behind this variation [15].

This study demonstrates the effect of *S. mansoni fs800* knockdown in adult females during pairing on the number of viable eggs produced *in vitro*. The knockdown led to 89.7 % and 92.6 % reduction in the number of viable eggs produced on days 36 and 45 dpi, respectively. In a similar study with *S. japonicum* a 50 % reduction in the number of eggs was observed in paired females after knockdown [21]. Part of the explanation for the difference could be due to the RNAi knockdown only resulted in a 60 % reduction in *Sjfs800* [21] whereas we achieved over 90 % reduction in *Smfs800*. Another explanation could be the higher rate of reduction in the number of viable eggs produced by worm pairs in our study, comes from the lower number of eggs produced by *S. mansoni* about 300 eggs per worm pair [7] compared to the number of eggs produced by *S. japonicum* about 1–2000 eggs per worm pair per day [37]. Therefore higher concentrations of RNAi might be needed to effect more significant knockdown.

Although the vitellaria from knockdown groups have well-developed vitelline cells with intact nuclei and cytoplasmic granules, the number of vitelline cells from knockdown females was significantly lower than the number of vitelline cells from control females. This might result in reducing the number of vitelline cells available to surround the ootype during egg formation and also result in less nutrient material [38]. Since *fs800* is not expressed in the ovary, it was not expected that the *fs800* knockdown would affect the growth and development of the female ovary. *Fs800* products may have other roles related to oogenesis other than an eggshell precursor [20]. For example, a considerable number of eggs have a defective eggshell among the non-viable eggs produced from all three RNAi treated groups (Fig. 2). Therefore, our data indicate that *fs800* products play a role in eggshell formation and due to the number of dead embryos likely have a role in nutrition for the embryo. Interestingly, the vitellaria subjected to RNAi knockdown, in addition to showing reduced numbers of vitellocytes also shows an increase in what appears to be released cytoplasmic granules which may speak to a role for vitellocyte integrity for *fs800*. Although apoptosis is known to occur as a mechanism to reduce vitellocyte numbers, the morphological evidence suggests that this is not the case [39]. The lack egg production was not due to the failure of the male to mate with the female as no significant changes in the number of pairing worms were observed in any of the knockdown groups compared to the control. This is in contrast to what was reported for *S. japonicum* [21].

In conclusion, this study demonstrates that *fs800* plays an important role in eggshell formation, perhaps nutrients for embryo development and maintaining the integrity of vitellocytes.

## Supplementary Material

Supplementary
