# Supplementary material for: The effect of fs800 on female egg production in Schistosoma mansoni
Source: Mol Biochem Parasitol. Author manuscript; Available in PMC 2024 Feb 3. (PMC10838108; doi:10.1016/j.molbiopara.2021.111412)
Supplement: Supplementary [file NIHMS1960748-supplement-Supplementary.docx]

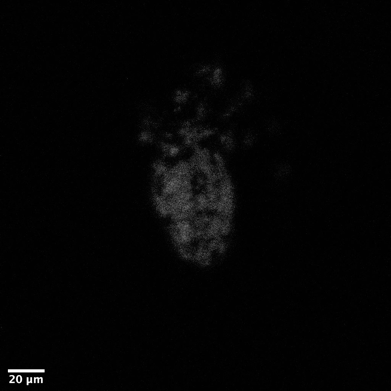

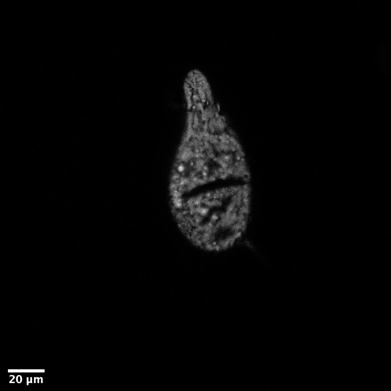
**Supplementary figure 1**

**A B**

Videos for eggs collected from *in vitro* cultured 45 dpi worm pairs using Zeiss 710 confocal microscope. **A.** Egg collected from control. **B** Egg collected from *Smfs800* knockdown.

**Supplementary figure 2**


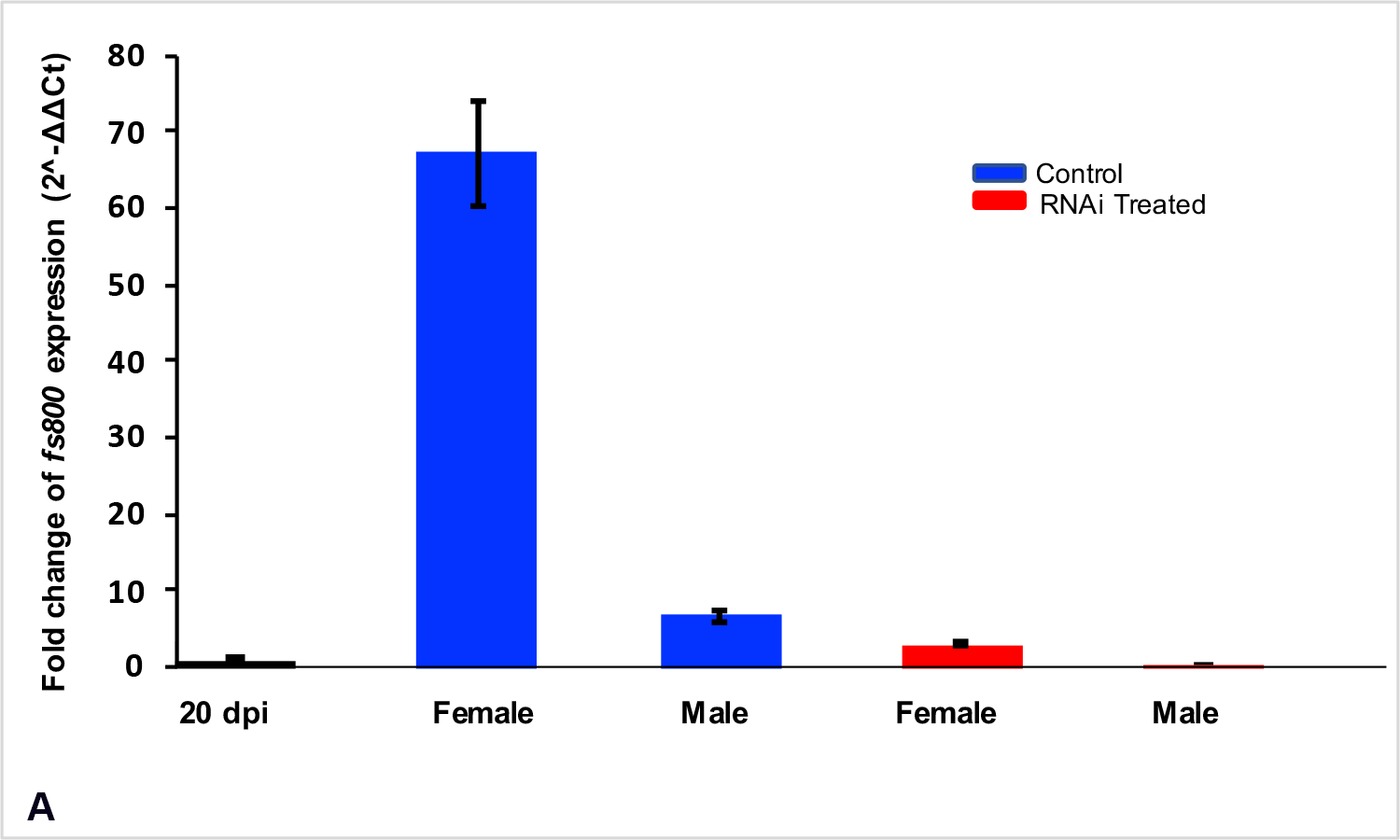

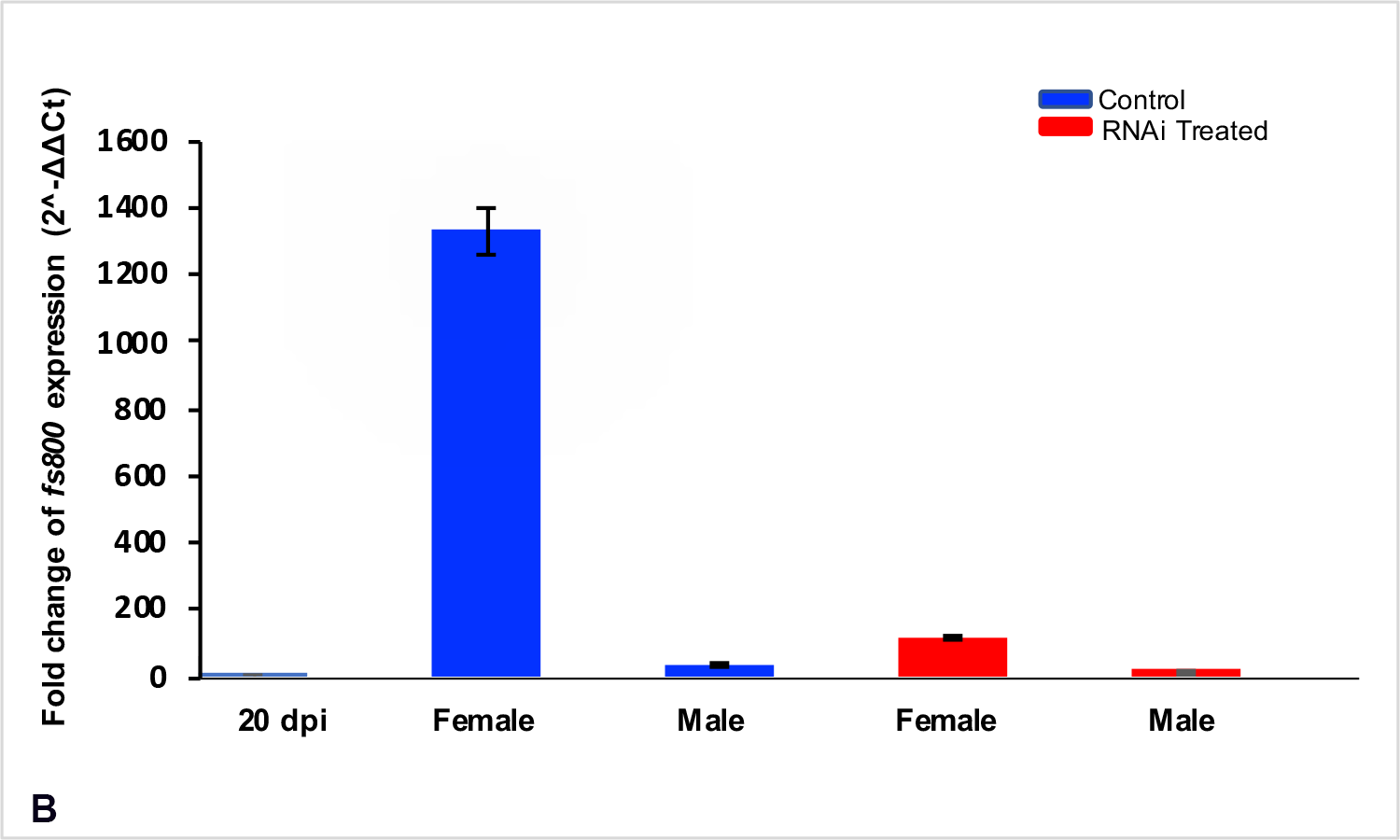


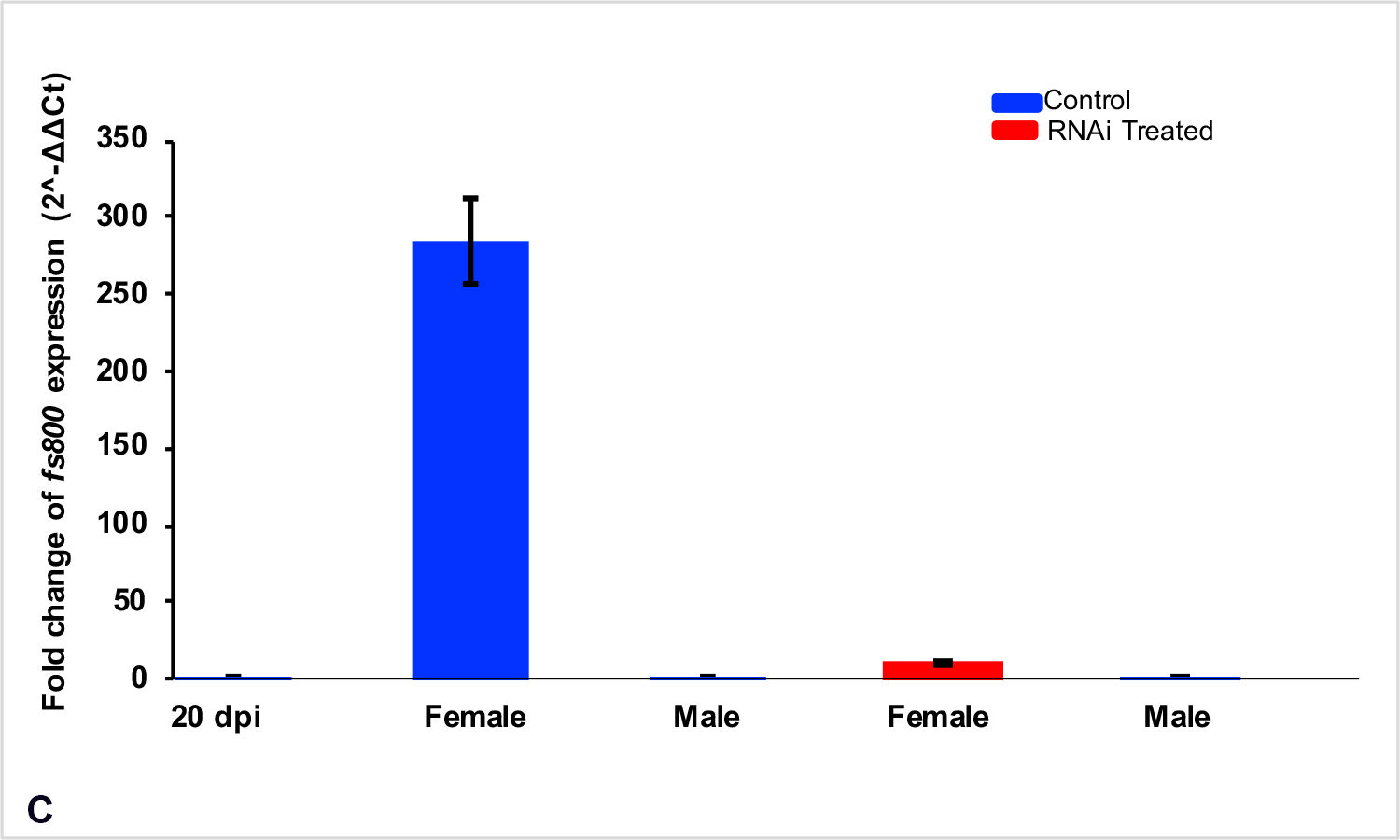


**S**upplemental **Figure2: *Smfs800* expression in RNAi treated worms and controls by qRT-
PCR. A**. *Smfs800* expression in 29 dpi. **B**. *Smfs800* expression in 36 dpi. **C.** *Smfs800* expression in 45 dpi. Transcripts of the *Smfs800* gene from male and female *S. mansoni* from RNAi treated worms and controls were evaluated by quantitative reverse transcriptase PCR. GAPDH was used as an internal reference and relative quantities were determined by the ΔΔCt method. Data represent the average of three independent experiments compared to the 20 dpi. Raw Ct values were analyzed in Excel.

**Supplemental Table 1**: The number of eggs exhibiting defective eggshells and dead embryos among non-viable eggs collected after 72 hrs from RNAi treated worms compared to control worms

|  | Total number of eggs produced (viable+ non-viable)/ Control worms | Total number of eggs produced (viable+ non-viable) | Total number of non-viable eggs | Non-viable eggs have defective eggshell | Non-viable eggs have dead embryo |
| --- | --- | --- | --- | --- | --- |
| 29 dpi | 1.0 × 10^4^ | 0.4 × 10^4^ | 0.3 × 10^4^ | 0.2 × 10^4^ | 0.1 × 10^4^ |
| 36 dpi | 3.55 × 10^4^ | 0.9 × 10^4^ | 0.55 × 10^4^ | 0.2 × 10^4^ | 0.35 × 10^4^ |
| 45 dpi | 2.72 × 10^4^ | 0.6 × 10^4^ | 0.4 × 10^4^ | 0.2 × 10^4^ | 0.2 × 10^4^ |

*The number is a total of triplicate wells
